# Contribution of Evolutionary Selected Immune Gene Polymorphism to Immune-Related Disorders: The Case of Lymphocyte Scavenger Receptors *CD5* and *CD6*

**DOI:** 10.3390/ijms22105315

**Published:** 2021-05-18

**Authors:** Sergi Casadó-Llombart, María Velasco-de Andrés, Cristina Català, Alejandra Leyton-Pereira, Francisco Lozano, Elena Bosch

**Affiliations:** 1 Immunoreceptors del Sistema Innat i Adaptatiu, Institut d’Investigacions Biomèdiques August Pi i Sunyer, 08036 Barcelona, Spain; secasado@clinic.cat (S.C.-L.); mvelascod@clinic.cat (M.V.-d.A.); catala@clinic.cat (C.C.); leyton@clinic.cat (A.L.-P.); 2 Servei d’Immunologia, Hospital Clínic de Barcelona, 08036 Barcelona, Spain; 3 Departament de Biomedicina, Universitat de Barcelona, 08036 Barcelona, Spain; 4 Department of Experimental and Health Sciences, Institute of Evolutionary Biology (UPF-CSIC), Universitat Pompeu Fabra, 08003 Barcelona, Spain; 5 Centro de Investigación Biomédica en Red de Salud Mental (CIBERSAM), 43206 Reus, Spain

**Keywords:** scavenger receptors, *CD5*, *CD6*, immune response, cancer, autoimmunity, infections, natural selection, human genetics, single nucleotide polymorphisms

## Abstract

Pathogens are one of the main selective pressures that ancestral humans had to adapt to. Components of the immune response system have been preferential targets of natural selection in response to such pathogen-driven pressure. In turn, there is compelling evidence showing that positively selected immune gene variants conferring increased resistance to past or present infectious agents are today associated with increased risk for autoimmune or inflammatory disorders but decreased risk of cancer, the other side of the same coin. *CD5* and *CD6* are lymphocytic scavenger receptors at the interphase of the innate and adaptive immune responses since they are involved in both: (i) microbial-associated pattern recognition; and (ii) modulation of intracellular signals mediated by the clonotypic antigen-specific receptor present in T and B cells (TCR and BCR, respectively). Here, we review available information on *CD5* and *CD6* as targets of natural selection as well as on the role of *CD5* and *CD6* variation in autoimmunity and cancer.

## 1. Evolution and Selective Pressure on Immune Receptor Genes: Examples of Selection

### 1.1. Human Evolution and Pathogens

Since our origin and migration out of Africa, humans have colonized many new environments and encountered different types of selective pressures to which we have adapted. Pathogens are recognized as one of the strongest selective agents that humans have faced through our recent evolutionary history. Especially from the Neolithic transition, infectious diseases have greatly influenced our innate and adaptive immune defense systems. While epidemic infectious diseases could probably not sustain themselves efficiently in hunter-gatherer small groups, evidence suggests that they started to cause major effects on sedentary and overcrowded agricultural sites [1]. In turn, animal domestication in Neolithic sites facilitated close contact with animals and higher risk for zoonoses. Several diseases such as malaria, measles, tuberculosis and smallpox are thus likely to have spread with this cultural and environmental shift. Despite the huge health improvements facilitated by the discovery of antibiotics and the development of modern vaccination programs, several recent epidemic outbreaks, such as those caused by several coronaviruses, the Zika virus or Ebola, reminds us, even nowadays, of the importance of our immune response to pathogens and how such infectious agents continue to exert important health pressures among humans [2].

At a local scale, our past adaptation to these pathogen-driven selective pressures has generated important differences in our immune response across modern human populations that often explain local differential susceptibilities to autoimmune, inflammatory-related traits and cancer [3]. Moreover, the enrichment of signatures of positive selection found in loci associated with common inflammatory disorders [4] has been interpreted to support the hygiene hypothesis, which states that the increasing incidence of both autoimmune and allergic disorders we observe today will be partly due to the huge contrast between the environmental pathogen load in which our immune system evolved and the more sterile world in which modern societies live today [5]. Within this context, the identification of genomic signatures of natural positive selection that are related to our immune system is the first step to not only elucidate potential adaptations to pathogen exposure but also to identify functional variation affecting a wide range of immune-related phenotypes.

### 1.2. Detecting Local Adaptation in the Human Genome

Natural selection leaves distinctive footprints or signatures in the patterns of variation around adaptive genetic variants, which can be detected when compared with background genome-wide patterns of variation in the genome and/or by considering demographic simulations of populations. The development of new statistical tools and approaches for the detection of selection in the recent years, together with the increasing availability of large catalogues of genetic variability in different human populations, has allowed the identification of hundreds of loci with signatures of selection in our genome [6,7,8,9]. Most genome-wide scans of positive selection in humans have focused on detecting the selection signatures expected from hard sweeps, where a new mutation is favored and raises rapidly its frequency, sweeping its linked variation. In such a scenario, several statistical tools using intraspecific variation have been developed to capture high population differentiation, site frequency spectrum skews and unusually long-range linkage disequilibrium [7]. However, over the last few years, new strategies have also been developed to capture the patterns of variation of the soft sweeps resulting from either multiple de novo mutations or from standing variation, as well as on detecting polygenic selection and adaptive introgression [10]. Multiple immune-related genes have been reported as candidates for positive selection when applying these methodologies [4]. However, for most of the detected signatures, the individual infectious agents driving the selective pressures are not well understood, neither are the underlying adaptive variants and immunological adaptive responses associated to them. Multidisciplinary strategies integrating functional annotations in our genome such as those available in ENCODE, with results from genome-wide association studies (GWAS), expression quantitative trait loci (eQTLs), cell count quantitative trait loci [11], cytokine quantitative trait loci (cQTLs) [12], immune-responsive regulatory variation [13], in silico predictions from protein structure modeling [14,15], etc. [16] have been shown to facilitate the identification of adaptive variants related to our immune response.

### 1.3. Examples of Positive Selection at Immune Response Receptors

The mammalian immunity relies on receptors from both the innate and adaptive immune systems. The innate immune receptors—also called pattern-recognition receptors (PRRs)—are adapted to the recognition of conserved and broadly shared components of microbial surfaces, which are absent from the host and are essential for microbial viability—the so-called pathogen-associated molecular patterns (PAMPs). There are several functionally distinct classes of PRR, which can be expressed in either secreted or membrane-bound form by innate immune cells (macrophages, dendritic cells or natural killer cells). The best studied PRRs are the Toll-like receptors (TLRs), but additional relevant families include C-type lectin receptors, scavenger receptors, collectins, pentraxins, NOD-like receptors (NLRs) or RIG-like receptors (RLRs), among others. The recognition in the adaptive immune system is mediated by highly polymorphic clonotypic receptors expressed by T and B cells (TCR and BCR, respectively). These receptors recognize highly specific microbial details, which in the case of T cells need to be presented by major histocompatibility complex (MHC) class I or II molecules. As expected, natural selection has acted on both the innate and the adaptive immune receptors, and we briefly discuss some examples.

The region encompassing a cluster of three TLRs in chromosome 4 (*TLR10*-*TLR1*-*TLR6*) provides a notable example of how our adaptation to microbes favored several parallel innate immune responses during our recent evolutionary history. Among others, a non-synonymous substitution (Ile602>Ser) on TLR1 has been shown to impair signaling with a drastic decrease of NF-κB activity and reported as a potential target of positive selection detected in the *TLR10-TLR1-TLR6* cluster in Europeans [17]. Convergent signals of very recent positive selection in European and Roma populations have also been suggested to result from the plague, since several non-synonymous changes on the *TLR10-TLR1-TLR6* cluster present in Europeans modulate *Yersinia pestis*-induced cytokine responses [18]. In addition, independent events of adaptive introgression from both Neanderthals and Denisovans in non-African populations have also been described in the *TLR10-TLR1-TLR6* region. Notably, the adaptive alleles resulting from these admixture events with archaic humans are associated with increased expression of TLR6, TLR1 and TLR10 in white blood cells, reduced *Helicobacter pylori* seroprevalence and increased susceptibility to allergies [19]. Whereas an impaired TLR-mediated response resulted beneficial in the case of the Ile602>Ser substitution on TLR1, the introgressed alleles of the *TLR10-TLR1-TLR6* cluster probably reinforced innate immune surveillance and reactivity against certain pathogens [19]. In any case, the selected functional variation resulting from these past local adaptive events, with or without the actual presence of the driving infectious agent, has undoubtedly the potential to influence many distinct inflammatory and allergenic susceptibilities across present-day populations.

It has been suggested that mutations causing deficiency of the scavenger receptor CD36 in African and East Asian populations may have been selected to protect against malaria [20]. For instance, patterns of extended haplotype homozygosity compatible with the action of recent positive selection and a frequency of up to 0.26 have been observed for a non-sense mutation in exon 10 of *CD36* (Thr1264>Gly; rs3211938) in west central Africans. Although no association with severe malaria was found in that case, alternative evolutionary scenarios were suggested to explain the prevalence of the CD36 deficiency caused by Thr1264>Gly [20]. Notably, CD36 is not only involved in immunological recognition and molecular adhesion but also in lipid metabolism, angiogenesis and metastasis in cancer [21]. Thus, adaptive variation on *CD36* facilitating host survival to pathogens could in turn influence a variety of traits and conditions. Within this context, CD36 deficiency has been shown to reduce atherosclerotic lesion formation [22] but also to cause dyslipidemia, subclinical inflammation and metabolic disorders [23].

The MHC is the most important region in the human genome influencing our response to infection, inflammation and autoimmunity. The extremely high levels of allelic diversity observed and the sharing of ancestral polymorphisms with other hominoid taxa at the MHC class I and II genes have been suggested to result from overdominant selection, a model in which heterozygote individuals have a higher biological fitness than the homozygotes [24]. However, frequency-dependent selection and selection that varies over time and space have also been proposed to act on the human leukocyte antigen (HLA) genes [25]. Interestingly, between-population variation at the HLA class I genes were found to be positively correlated with local pathogen richness (notably for the *HLA-B* gene), thus supporting the hypothesis that pathogen-driven selection may have created the high polymorphism levels observed in this MHC complex [26]. Notably, unusually high frequency extended haplotypes comprising variants associated with systemic lupus erythematosus (SLE), multiple sclerosis (MS) and type I diabetes were identified for the *HLA-DR2*, *HLA-DRB1* and *HLA-C* loci in samples of European descent [27], illustrating again how the identification of immune loci with signatures of recent positive selection may be a good strategy to identify functionally relevant variation and suggest candidates to test for association to immune related diseases [4,28]. Furthermore, single nucleotide polymorphisms (SNPs) on the HLA region associated to type I diabetes, SLE, psoriasis (Ps) and rheumatoid arthritis (RA) have also been shown to present values of the integrated haplotype score (iHS) statistic, indicative of recent positive selection in samples of European origin [4].

## 2. The Case of Lymphocyte Scavenger Receptors *CD5* and *CD6*

### 2.1. The CD5 and CD6 Protein Receptors: Structure and Function

The *CD5* and *CD6* receptors are type I transmembrane glycoproteins belonging to the scavenger receptor cysteine-rich superfamily (SRCR-SF). This is a functionally diverse superfamily of innate immune receptors characterized by the presence of one or several repeats of the ancient and highly conserved SRCR domain. Accordingly, both *CD5* and *CD6* proteins are composed by three tandem extracellular SRCR domains (from N- to C-terminal: SRCR1, SRCR2 and SRCR3), followed by a transmembrane domain and a cytoplasmatic tail devoid of enzymatic activity but well adapted for Thr/Ser/Tyr phosphorylation and intracellular signaling.

*CD5* and *CD6* are expressed by all T cell types and the B1a cell subset, with lower levels of expression in other cell types (e.g., macrophages, dendritic cells or natural killer cells) [29,30]. *CD5* and *CD6* are two functionally relevant co-receptors involved in the fine tuning of lymphocyte activation and differentiation upon specific antigen recognition [31]. Such immunomodulatory function is facilitated by their physical association to the clonotypic antigen-specific receptor complex of T (TCR) and B (BCR) cells and their ligation by endogenous counter receptors during cell-to-cell contacts. The reported *CD6* ligands include CD166/ALCAM (activated leukocyte cell adhesion molecule) [32,33,34,35,36], galectins 1 and 3 [37] and CD318/CDCP-1 (CUB domain containing protein 1) [38]. Interaction with CD166/ALCAM has been extensively studied, and it is known to involve the most membrane-proximal SRCR domain of *CD6* (SRCR3) and the most amino-terminal immunoglobulin V-like domain of CD166/ALCAM (V1). Such interaction is relevant to immune synapse stabilization [32,33,34,35,36] and lymphocyte transmigration [39]. As for *CD5*, there is a long list of reported ligands (e.g., CD72, IgVH framework region, gp150, IL-6 and *CD5* itself), none of which has been unequivocally confirmed by independent groups [40,41].

Upon *CD5* and *CD6* receptor ligation and phosphorylation, several downstream intracellular signal transducers are engaged. The multiple interactors recruited to the *CD5* signalosome (SHP-1, Ras-GAP, Cbl-b, UBASH3A, ANKRD13A and CK2) finally end up down-modulating lymphocyte activation [42,43,44,45,46]. As for the *CD6* signalosome, the reported interactors (SLP-76, ZAP-70, VAV1, GRB2, GRAP2, GRK, GADS, TSAd, UBASH3A, SHIP1, ARHGAP45 and Syntenin) end up providing co-inhibitory or co-stimulatory functions [46,47,48,49,50].

In recent times, the interaction of both receptors with PAMPs from bacterial, fungal, viral or parasitic origin has been reported. Particularly, *CD5* has been shown to interact with fungal β-glucans [51], hepatitis C virus [52] and tegumental structures of the *Echinococcus granulosus* parasite [53], while *CD6* can interact with lipopolysaccharide, lipoteichoic acid and peptidoglycan of Gram-positive and -negative bacteria [54], gp120 from human immunodeficiency virus 1 [55] and *E. granulosus* tegument components [53]. In line with this PRR function, mouse data indicate that *CD5* is a non-redundant integral component of host’s immune response to fungal infection [56], and our preliminary observations would indicate that this could be also the case for *CD6* regarding bacterial infection [57].

### 2.2. The CD5 and CD6 Genes: Location, Exon/Intron Organization and Isoforms

**Figure 1:**
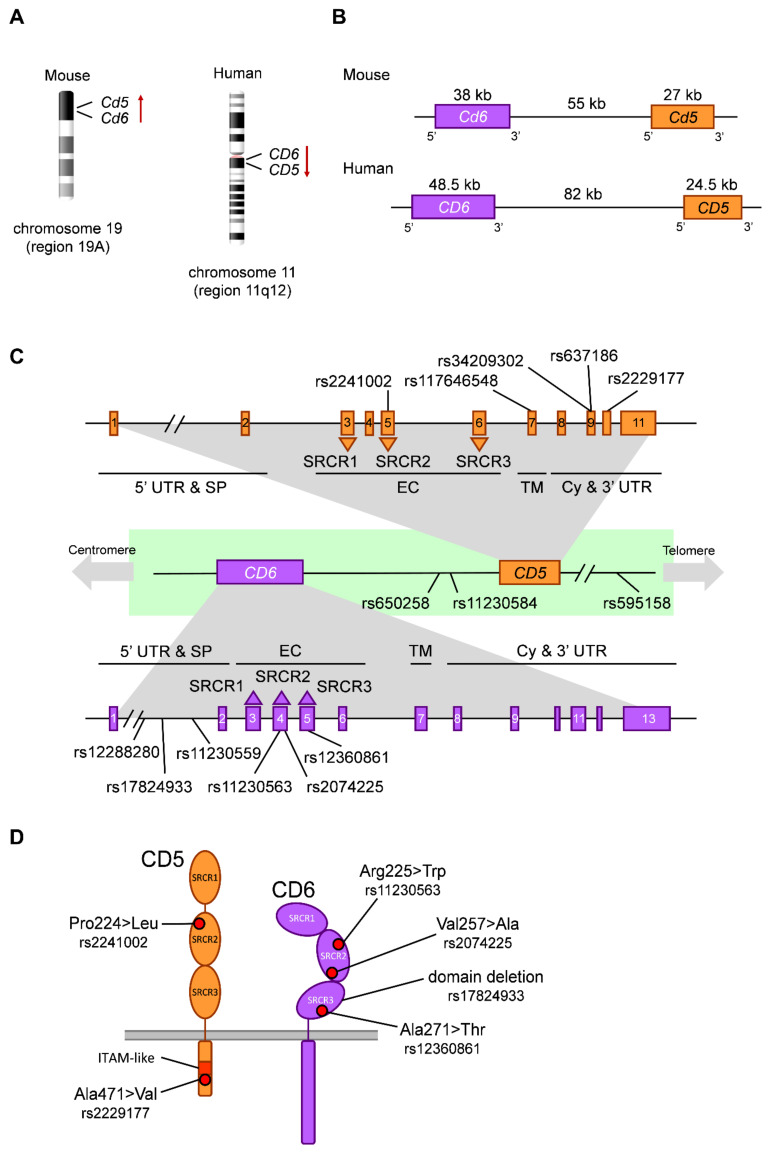
Genomic location and arrangement of *CD5* and *CD6*. (**A**) Chromosome location of mouse and human genes coding for *CD5* and *CD6*. Red arrows indicate 5′ to 3′ orientation. (**B**) Size, orientation and intergenic distance regarding *CD5* and *CD6* in mouse and human. (**C**) Exon/intron organization, protein coding regions and location of relevant SNPs in *CD5* and *CD6*. (**D**) Structure of membrane *CD5* and *CD6* showing the impact of relevant SNPs. UTR, untranslated region; EC, extracellular region; Cy, cytoplasmic region; SRCR, scavenger receptor cysteine-rich domain; ITAM-like, immunoreceptor tyrosine-based activation motif–like.

The *CD6* and *CD5* genes lie less than 100 kb apart in the long arm of human chromosome 11q12.2 and in the orthologous region of mouse chromosome 19 [58,59]. *CD5* consists of 11 exons encompassing a 24.5 kb region located 82 kb in 3′ direction to *CD6*, in a head-to-tail orientation (Figure 1). This, together with the high structural, functional and tissue expression similarity between the *CD5* and *CD6* receptors, leads to the assumption that both genes arose from duplication of an ancestral gene [58,59].

There is a good correlation between exon-intron organization and the structural domains of the *CD5* protein: each SRCR domain is encoded by an individual exon (exons 3, 5 and 6). The Pro/Thr-rich region that connects SRCR1 and SRCR2 domains is encoded by exon 4. The transmembrane domain is encoded by exon 7 and the cytoplasmic tail by exons 8, 9 and 10 [58,59] (Figure 1). The signal peptide is encoded by exons 1 (18 aa) and 2 (6 aa). Exon 1 also contains the untranslated 5′ region (5′-UTR). Finally, exons 10 and 11 contain the stop codon and the 3′-UTR region, respectively [58,59]. There are two polyadenylation signals in exon 11 that could explain the identification of two species of *CD5* mRNA (2.7 and 3.6 kb each) [60]. While for *CD6* several isoforms have been described resulting from alternative mRNA splicing, a *CD5* isoform encoding a protein with transmembrane region but no cytoplasmic tail has been detected from total peripheral blood mononuclear cell (PBMC) mRNA, for which no information is yet available on its expression pattern or function [58,59]. An alternate regulatory exon 1 (designated E1B) located ~8.2 kb upstream the ATG initiation codon of the conventional exon 1 (renamed E1A) of the human *CD5* gene has also been reported [61]. The E1B-containing transcripts exist exclusively in B lymphocytes and encode a truncated protein devoid of the leader peptide and retained intracellularly. As a consequence, the amount of E1A-containing transcripts is downregulated and the membrane *CD5* expression is diminished in the presence of E1B-containing transcripts [61].

The *CD6* gene consists of at least 13 exons, with the first 6 coding for the 5′-UTR, the signal peptide (exons 1 and 2), the three extracellular SRCR domains (exons 3–5) and the stalk region (exon 6) [62]. The transmembrane region is encoded by exon 7, the cytoplasmic region by exons 8–11 and the 3′-UTR region by at least exon 13 (Figure 1). As mentioned above, *CD6* undergoes alternative RNA splicing that result in isoforms devoid of the SRCR3 extracellular domain (*CD6*Δd3) or certain intracytoplasmic signaling motifs [62,63,64], which are defective in binding to CD166/ALCAM and proper cytoplasmic tail phosporylation and intracellular signaling, respectively.

### 2.3. Functionally Relevant CD5 and CD6 Polymorphisms

A long list of SNPs has been identified in the *CD5* and *CD6* loci, with some of them showing functional relevance (Table 1). Regarding *CD5*, the two most relevant SNPs reported to date are rs2241002 (C>T) and rs2229177 (C>T), which result in amino acid substitutions at the SRCR2 domain (Pro224>Leu) and just next to an ITAM-like cytoplasmic motif (Ala471>Val), respectively [65,66]. Cell transfectants expressing the Ala471 variant (rs2229177^C^) show lower mitogen-activated protein kinase (MAPK) activation and IL-8 production when crosslinked with anti-*CD5* monoclonal antibodies or exposed to the fungal β-glucan-rich particle Zymosan, respectively, with regard to the Val471 variant (rs2229177^T^) [65]. This would be compatible with the Ala471 variant having a lower signaling capability and, consequently, lower ability to negatively modulate the activation signals delivered by the clonotypic antigen-specific receptor complex. Accordingly, PBMCs from homozygous Ala471 (rs2229177^CC^) donors show higher T-cell proliferative responses than homozygous Val471 (rs2229177^TT^) donors [66]. An interpretation for these observations is that the ancestral *CD5* variant Ala471 has a lower negatively modulatory capacity of the TCR than the Val471 variant.

Regarding *CD6*, the list of identified SNPs includes the non-synonymous rs11230563 (C>T) and rs2074225 (T>C) SNPs causing amino acid substitutions at the SRCR2 domain (Arg225>Trp and Val257>Ala, respectively) and the rs12360861 (G>A) at SRCR3 domain (Ala271>Thr). Intronic *CD6* SNPs include rs12288280 (G>T), rs17824933 (C>G) and rs11230559 (T>C) in intron 1, together with the 3′ intergenic SNP rs650258 (T>C) [67]. Efforts to unveil the effect of these variants in the *CD6* function show that the rs17824933^G^ allele is associated to skipping of exon 5, resulting in increased expression of a *CD6* isoform lacking the SRCR3 domain (*CD6*Δd3), in which the CD166/ALCAM-binding site is located [64,68]. Although this does not result in a change of the total *CD6* amount on the cell surface, increased Δd3/full-length *CD6* ratio results in lower activation of CD4^+^ lymphocytes [68]. No direct impact on *CD6* expression or function has been described yet for rs11230559, but it has been shown to be in linkage disequilibrium with rs17824933 and the non-synonymous SNPs rs11230562 in SRCR2 (C>T; Thr217>Met) and intracellular rs2074233 (G>A, Gly606>Ser) [67]. The *CD6* haplotype involving rs11230563^C^ and rs2074225^C^ SNPs (Arg225 Ala257) results in higher *CD6* surface expression on several lymphocyte subsets (CD4^+^ and CD8^+^ naïve T cells and NKT cells) [67]. Quantitative trait loci studies have shown that the rs11230584 SNP in the intergenic region between *CD5* and *CD6* modulates expression of both genes under certain pathological circumstances [69].

### 2.4. Discovery of the CD5 and CD6 Loci as Targets of Natural Selection

Using SNP genotyping data, we previously described signatures of recent positive selection for several East Asian populations along a genomic region of 0.5 Mb on chromosome 11 comprising several genes besides *CD5* and *CD6* [70]. In particular, we generated SNP genotyping data in the worldwide HGDP-CEPH diversity panel and explored for extreme population differentiation, excess of low-frequency variants and high frequency-derived alleles, as well as for long-range haplotypes. Whereas no signals of positive selection were found outside East Asia for this genomic region, all East Asian populations displayed the expected pattern of genetic variation of a classical selective sweep. Notably, at least six other genome-wide scans for positive selection have recognized a strong signal for a recent selective sweep in East Asia in the same region [5]. By analyzing all potential functional SNP data linked to detected signatures, in our original study, we already suggested a non-synonymous SNP (rs2229177) on the *CD5* gene as the putative target of selection [70]. Subsequently, we further confirmed deviations from neutrality in the Chinese population using Sanger sequencing data on the *CD5* gene and demonstrated the functional relevance of the Ala471>Val substitution (rs2229177) [65]. Thus, we suggested that the different signaling capabilities observed for the Ala and Val alleles might have driven the signals of selection previously detected in East Asians [65].

Most signatures of recent positive selection usually comprise large genomic regions with many different potential putative adaptive genes and variants. Without a priori adaptive hypotheses or known selective pressures, the subsequent identification of the true adaptive genes and variants driving each selection signal in a genome-wide scan is not direct but a laborious endeavor. The *CD5* gene is less than 100 kb apart from *CD6* and near the *VPS37C* gene, which encodes for a component of the endosomal sorting complex required for transport I (ESCRIT-I) and important for viral budding [71]. Any of these three genes could have facilitated geographically local adaptation related to our immune response in East Asian populations. The presence of two recombination hotpots between the *CD5* and *CD6* genes together with the identification of most signatures of recent positive selection in East Asians extending towards the *CD5* and *VPS37C* genes (but not always towards *CD6*) seem to point that probably any linked variation in *CD5* and *VPS37C* could have resulted adaptive and created such local pattern of selection. Since rs2229177 was the only non-synonymous SNP with in silico-predicted phenotypical effects and the allele frequencies for the derived Val471 variant matched the detected signatures of directional selection and characterized a major haplotype found in East Asia, it was first suggested as the putative target of selection [70] and then functionally validated [65]. Unfortunately, no specific pathogen or infectious disease was recognized as the selective force driving such local selective event.

Although not linked to signals of recent positive selection, the two *CD5* derived alleles at rs2229177 and at rs2241002 (i.e., the Val471 and Leu224 variants) are present at intermediate frequencies in Africa (0.51 and 0.31) and Europe (0.55 and 0.15). Thus, they probably determine not only potential differential immune responses to pathogens but also important immune-related susceptibilities among their carriers. Similarly, two non-synonymous SNPs in the *CD6* gene (rs11230563 and rs2074225) that are predicted to be deleterious (with CADD scores of 22.4 and 17.66, respectively) display high heterozygosities in Africa (with derived allele variants of 0.61 and 0.33, respectively), Europe (0.36 and 0.38) and America (0.30 and 0.56). In contrast to the directional selective event described in East Asia that led to the fixation of the Val471 variant, the intermediate allele frequencies found at these *CD5* and *CD6* functional relevant polymorphisms could have been favored by balancing selection maintaining genetic polymorphism and providing functional versatility at these immunity genes.

### 2.5. CD5 Polymorphism in Autoimmunity and Cancer

Functionally relevant *CD5* SNPs have been investigated as putative susceptibility or disease modifier markers in autoimmune and neoplastic disorders. Accordingly, GWAS has shown association of *CD5* variation (rs595158) with RA susceptibility [71]. Association studies of *CD5* variation in SLE show that the rs2241002^C^ (Pro224) and rs2229177^C^ (Ala471) alleles are associated with the development of lupus nephritis [65], which represents a severe form of the disease. The same study showed that the rs2241002^C^-rs2229177^C^ haplotype (Pro224-Ala471) is overrepresented in SLE patients with nephritis. This finding would agree with the reported lower negative immunomodulatory properties of the *CD5* Pro224-Ala471 variant (see Section 2.3). Our recent clinical association studies in patients afflicted of inflammatory bowel diseases (IBD) also anticipates association of *CD5* variation with location (rs2241002^CC^) and requirement of biological therapies (rs2241002^C^-rs2229177^T^ haplotype; Pro224-Val471) in Crohn disease, (CD) and with poor prognosis (rs2241002^T^-rs2229177^T^ haplotype; Leu224-Val471) in ulcerative colitis (UC) [72]. This would indicate that *CD5* variation differentially influences clinical outcomes depending on disease-specific etiopathogenic factors.

The inhibitory function of *CD5* in T and B1a cell activation has positioned this receptor as a relevant player in the immune response against cancer [40,41]. This is illustrated by studies on *CD5* variation in human malignancies. Thus, the rs2229177^C^ (Ala471) and the rs2241002^C^ (Pro224) alleles correlate with better outcome and increased melanoma-associated mortality, respectively [73]. This could be attributed again to the lower capacity of the rs2229177^C^ (Ala471) allele to downregulate activating TCR-mediated intracellular signals, which would potentiate T-cell anti-melanoma immune responses.

Apart from tumor infiltrating lymphocytes, *CD5* can also be expressed on certain malignant cells. There, the different signaling capabilities of *CD5* variants could play a role in their biological and/or clinical behavior. This is the case of chronic lymphocytic leukemia (CLL), the most frequent hematological malignancy in western countries [74]. Our clinical association studies show that CLL patients either homo- (rs2229177^CC^) or heterozygous (rs2229177^CT^) for the ancestral Ala471 allele present higher progression-free survival in the most prevalent but less aggressive subgroup of IGVH-mutated CLL [75]. This would indicate that *CD5* is not only a phenotypical marker but a relevant player in the biological or clinical behavior of CLL.

### 2.6. CD6 Polymorphism in Autoimmunity and Cancer

Several *CD6* SNPs have been associated to immune-mediated inflammatory disorders, including MS, Ps and Behçet’s disease. *CD6* is a consolidated risk locus for MS as stated by a meta-analysis of six GWAS [76]. This study identified the *CD6* rs17824933 SNP as a risk marker for MS in cohorts of European origin, with the rs17824933^G^ allele being associated to greater MS risk [76]. Further gene-specific approaches were then performed to confirm this observation. Association of rs17824933^G^ allele with increased MS risk was confirmed in twelve independent European cohorts [77,78]. A study aiming at fine mapping the *CD6* locus in MS in a European cohort [67] found an association of the rs2074225^T^ (Val257) allele with higher MS risk. Haplotypic analyses also found similar strong association for the *CD6* rs11230563^T^-rs2074225^T^ haplotype (Trp225-Val257), which involves non-synonymous substitutions at *CD6* SRCR2. In a mechanistic exploration, the risk haplotype rs11230563^T^-rs2074225^T^ correlated with lower *CD6* expression in various lymphocyte subsets (see Section 2.3) [67]. The same study also found association of rs11230559^C^ with higher MS risk and confirmed the risk alleles rs17824933^G^ and rs650258^C^ [79]. In another study, the *CD6* rs12360861^G^ (Ala271) allele was also associated to increased MS risk in a European cohort [80]. The same authors also found evidence for association between risk and progression of MS with variation at *CD166/ALCAM*. Such investigation revealed that individuals carrying the rs6437585^T^ allele had higher risk of MS and earlier age of onset [81]. Interestingly, in vitro studies show increased *CD166/ALCAM* transcriptional activity for the rs6437585^T^ allele [82], which would agree with investigations showing upregulated CD166/ALCAM expression on central nervous system vessels in active MS lesions [39].

*CD6* association studies in MS have also been performed in non-European cohorts. An African American cohort did not confirm association with the intronic rs17824933 SNP, but found the *CD6* SNP rs11230563^C^ (Arg225) as a risk marker for MS [83]. A replication study in an Asian cohort did not show any association of *CD6* SNPs with MS risk but found association of the intronic rs12288280^G^ allele with neuromyelitis optica, a similar demyelinating disease with distinct pathophysiology [84].

Regarding other inflammatory diseases, the *CD6* rs12360861^G^, rs17824933^G^ and rs11230563^C^ alleles have been found associated to increased Ps severity in a European cohort [85]. In Chinese Han population, rs11230563^T^ was found associated to increased risk of Behçet’s disease [86]. GWAS and meta-analyses also showed association between *CD6* rs11230563 SNP and susceptibility to IBD [87,88]. This would agree with our recent observations on *CD6* variation association with location (rs17824933^G^) and poor prognosis (rs12360861^G^) in CD patients and with left-sided or extensive UC (rs17824933^G^) [72].

As far as we know, there is no current evidence linking *CD6* expression and/or variation with susceptibility or prognosis to malignancies. This contrasts with the high number of studies reporting association of CD166/ALCAM expression with grade, stage and invasiveness of different carcinomas [89]. The known relevance of *CD6*-CD166/ALCAM interaction in cell-to-cell adhesive contacts established between T cells and other immune (B cells, macrophages, dendritic cells) and non-immune (endothelial, epithelial) cells warrants future studies of *CD6* variation in cancer.

**Table 1:** Reported frequency of functionally and clinically relevant *CD5* and *CD6* SNPs.

Gene	SNP	Alleles *	Change	CADD	AFR	EUR	EAS	SAS	AMR	Functional/Clinical Relevance
*CD5*	rs2241002	C>T	Pro224>Leu	11.06	0.31	0.15	0.06	0.15	0.14	T allele associated to lower risk of lupus nephritis [66] and higher melanoma mortality [73]. Haplotypic combinations with rs2229177 associated to lupus nephritis [66] and survival in melanoma [73] and chronic lymphocytic leukemia (CLL) [75].
	rs117646548	G>A	Ala377>Thr	11.86	0.00	0.01	0.00	0.00	0.01	
	rs34209302	C>T	His461>Tyr	0.092	0.08	0.01	0.00	0.08	0.01	
	rs637186	G>A	Arg461>His	0.014	0.01	0.08	0.00	0.04	0.05	
	rs2229177	C>T	Ala471>Val	25.2	0.51	0.55	0.99	0.80	0.66	T allele associated to more signaling upon *CD5* stimulation [65], stronger TCR inhibition [66], decreased lupus nephritis risk [66] and lower survival in melanoma [73] and CLL [75].
Inter-genic	rs650258	T>C		0.051	0.65	0.63	0.88	0.76	0.76	C allele associated to increased multiple sclerosis (MS) risk [67,79].
	rs11230584	G>A		1.789	0.25	0.15	0.13	0.18	0.11	Modulation of *CD5* and *CD6* expression [69].
	rs595158	C>A		2.165	0.55	0.54	0.99	0.79	0.67	Risk locus in rheumatoid arthritis [90].
*CD6*	rs12288280	G>T	Intronic	2.973	0.50	0.10	0.10	0.05	0.14	T allele associated to decreased neuromyelitis optica risk in an Asian cohort [84].
	rs17824933	C>G	Intronic	7.58	0.01	0.23	0.03	0.07	0.12	G allele associated to increased expression of *CD6*Δd3 [68], increased MS risk in European cohorts [76,77,78] and increased psoriasis severity [85].
	rs11230559	T>C	Intronic	4.239	0.01	0.25	0.04	0.07	0.12	In linkage disequilibrium with rs17824933 [67].
	rs11230563	C>T	Arg225>Trp	22.4	0.61	0.36	0.17	0.21	0.30	Haplotypic combinations with rs2074225 associated to differential *CD6* expression [67]. T allele associated to decreased MS risk in an African American cohort [83], decreased psoriasis severity [85] and increased Behçet’s disease risk in a Han population [86]. Involvement in inflammatory bowel disease [87,88].
	rs2074225	T>C	Val257>Ala	17.66	0.33	0.38	0.59	0.54	0.56	Haplotypic combinations with rs11230563 associated to differential *CD6* expression [67]. T allele associated to increased MS risk in a European cohort [67].
	rs12360861	G>A	Ala271>Thr	0.001	0.04	0.19	0.00	0.05	0.12	A allele associated to decreased MS risk in a European cohort [80] and increased psoriasis severity [85].

* Ancestral > derived. CADD, combined annotation-dependent depletion. Derived allele frequencies in populations from 1000 Genomes Project Phase 3 (AFR, African; EUR, European; EAS, East Asian; SAS, South Asian; AMR, American).

## 3. Concluding Remark

Pathogens have exerted strong selective pressures during human evolution, shaping human immunogenetics. This has resulted in the selection of genetic variants affecting immune function. *CD5* and *CD6* are multifaceted lymphocyte scavenger receptors, combining roles as immune response modulators and pattern recognition receptors. As such, evolutionarily selected and/or functionally relevant polymorphisms in the *CD5* and *CD6* loci have been shown to impact a wide variety of immune-related disorders such as autoimmunity and cancer, often considered two sides of the same coin. This not only reflects the relevance of genetic variation in the immune function, but also positions *CD5* and *CD6* as potentially useful diagnostic and prognostic disease markers, as well as targets of immunomodulatory therapies.

## Data Availability

The study did not report any data.
